# Effects of live yeast on differential genetic and functional attributes of rumen microbiota in beef cattle

**DOI:** 10.1186/s40104-019-0378-x

**Published:** 2019-09-04

**Authors:** Ibukun M. Ogunade, Jerusha Lay, Kenneth Andries, Christina J. McManus, Frederick Bebe

**Affiliations:** College of Agriculture, Communities, and the Environment, Kentucky State University, Frankfort, KY 40601 USA

**Keywords:** Functional potential, Live yeast, Rumen

## Abstract

Several studies have evaluated the effects of live yeast supplementation on rumen microbial population; however, its effect on differential microbial genes and their functional potential has not been described. Thus, this study applied shotgun metagenomic sequencing to evaluate the effects of live yeast supplementation on genetic and functional potential of the rumen microbiota in beef cattle. Eight rumen-cannulated Holstein steers were randomly assigned to two treatments in a cross-over design with two 25-day experimental periods and a 10-day wash-out between the two periods. The steers were housed in individual pens and fed 50% concentrate-mix and 50% red clover/orchard hay *ad libitum*. Treatments were (1) control (CON; basal diet without additive) and (2) yeast (YEA; basal diet plus 15 g/d of live yeast product). Rumen fluid samples were collected at 3, 6, and 9 h after feeding on the last d of each period. Sequencing was done on an Illumina HiSeq 2500 platform. Dietary yeast supplementation increased the relative abundance of carbohydrate-fermenting bacteria (such as *Ruminococcus albus*, *R*. *champanellensis*, *R. bromii*, and *R. obeum*) and lactate-utilizing bacteria (such as *Megasphaera elsdenii*, *Desulfovibrio desulfuricans*, and *D. vulgaris*). A total of 154 differentially abundant genes (DEGs) were obtained (false discovery rate < 0.01). Kyoto Encyclopedia of Genes and Genomes (KEGG) annotation analysis of the DEGs revealed that 10 pathways, including amino sugar and nucleotide sugar metabolism, oxidative phosphorylation, lipopolysaccharide biosynthesis, pantothenate and coenzyme A biosynthesis, glutathione metabolism, beta-alanine metabolism, polyketide sugar unit biosynthesis, protein export, ribosome, and bacterial secretory system, were enriched in steers fed YEA. Annotation analysis of the DEGs in the carbohydrate-active enzymes (CAZy) database revealed that the abundance of genes coding for enzymes belonging to glycoside hydrolases, glycosyltransferases, and carbohydrate binding modules were enriched in steers fed YEA. These results confirm the effectiveness of a live *S. cerevisiae* product for improving rumen function in beef steers by increasing the abundance of cellulolytic bacteria, lactic acid-utilizing bacteria, and carbohydrate-active enzymes in the rumen.

**Electronic supplementary material:**

The online version of this article (10.1186/s40104-019-0378-x) contains supplementary material, which is available to authorized users.

## Background

Live yeast products containing *Saccharomyces cerevisiae* are feed additives used to alter rumen fermentation for improved performance, health, and feed efficiency of ruminants [1]. Yeast products favor the growth of fiber-degrading bacteria, stabilize rumen pH by stimulating the population of lactate-consuming bacteria, and increase abundance of rumen microbes by reducing the rumen’s redox potential [1].

Given the role of live yeast product on rumen function, earlier studies utilized culture-based methods to assess the effects of live yeast in the rumen [2]; however, this technique is unable to cultivate most naturally occurring species in the rumen [3]. In recent years, several 16S ribosomal ribonucleic acid (rRNA) gene-based sequencing studies have advanced our knowledge of ruminal bacterial diversity and have reported the effects of *S. cerevisiae* products in the rumen [4]. However, 16S rRNA gene sequencing reveals the bacterial diversity without any insight into the functional potential of microbial communities [5]. In our recent companion study, belonging to the same feeding experiment as the current study [6], we integrated 16S rRNA gene sequencing and liquid chromatography-mass spectrometry-based metabolomics. Our results revealed that some bacterial genera that responded to treatment with a live *S. cerevisiae-*based additive showed positive correlations with metabolites involved in amino acid metabolism and biosynthesis as well as metabolism of energy substrates. Therefore, this study applied a shotgun metagenomic sequencing approach to offer taxonomic classification of sequences at the species level and reveal, for the first time, the effects of live yeast supplementation on differentially abundant genes and their functional potential.

## Methods

All experimental animals were managed according to guidelines approved by the Institutional Animal Care and Use Committee of Kentucky State University (protocol number 18–001). Detailed information about cows and feeding have been reported in our companion paper [6]. Briefly, eight rumen-cannulated Holstein steers were assigned randomly to one of two treatments in a cross-over design with two 25-day experimental periods and a 10-day wash-out between periods. The steers were housed in individual pens and fed 50% concentrate-mix and 50% red clover/orchard hay *ad libitum*. Mineral mix (Hubbard Feeds; Mankato, MN) was fed free-choice. Dietary treatments were (1) control (CON; basal diet without additive) and (2) yeast (YEA; basal diet plus 15 g/d of Peloton live yeast product; PMI, Arden Hills, MN, USA). The yeast additive was top-dressed on the concentrate mix from d 1 to 25 of each period.

### Rumen fluid collection

On d 25 of each experimental period, representative samples (100 mL) of the ruminal contents were collected via the cannula at approximately 3, 6, and 9 h after feeding. At the time of collection, the samples were hand-strained through 4 layers of sterile cheesecloth to separate the liquid and solid samples. Daily composited samples (solid and liquid samples) were mixed 1:1 (*w*/*w*) and stored at − 80 °C for subsequent shotgun metagenomic sequencing.

### Shotgun metagenomic sequencing

Deoxyribonucleic acid (DNA) extraction and sequencing were performed as reported by Ogunade et al. [7]. Briefly, rumen fluid samples were thawed at room temperature and centrifuged at 15,000×*g*. DNA was extracted and purified from the pellets using a PowerSoil DNA Isolation Kit (MO BIO Laboratories Inc., Carlsbad, CA). The integrity of the DNA was verified by agarose (0.7%) gel electrophoresis and the DNA was stored at − 20 °C until further use. The sequencing library was constructed by DNA fragmentation [8]. The sheared DNA fragments were then sequenced on an Illumina HiSeq 2500 platform using an Illumina HiSeq – paired-end 150 base pair (bp) strategy. Quality control was performed at each step of the procedure to guarantee the reliability of the sequence data.

### Bioinformatics and statistical analysis

The raw sequence data files were demultiplexed and stored as fastq format. Quality filtering, such as removal of low-quality (Q-score ≤ 10) and contaminative reads, was performed. Thereafter, the quality-filtered sequence data were assembled using Iterative De Bruijn Graph De Novo Assembler [9]. The resulting contig sequences (≥ 500 bp) were evaluated using Quality Assessment Tool for Genome Assemblies (QUAST) software [10]. Thereafter, the contig sequences were aligned to National Center for Biotechnology Information’s reference sequence database using Kraken software [11] to obtain taxonomic composition and relative abundance information. Differences in the relative abundance of taxa at the species level (> 0.1% of total sequences) were analyzed using the generalized linear mixed model procedure in SAS version 9.4 (SAS Institute Inc., Cary, NC, USA); the model included the effects of treatment, period, and their interaction. Differences between means were determined using the Fisher’s test and significant differences were declared at *P* ≤ 0.05 and tendency was declared at 0.05 < *P* ≤ 0.10. Normality was tested by examining the distribution of residuals.

The open reading frames in the assembled contigs were predicted using GeneMark software [12]. Redundancy was removed and all predicted genes were clustered using Cluster Database at High Identity with Tolerance (CD-HIT) software (http://www.bioinformatics.org/cd-hit/) with the following parameters: 90% similarity, 90% coverage threshold [13]. Differential gene abundance analysis between CON and YEA was performed using DESeq [14]. Fold change (the ratio of abundance between CON and YEA treatments) ≥ 2 and false discovery rate (FDR) < 0.01, obtained using the Benjamini-Hochberg calibration method, were set as screening criteria to obtain differential gene sets. The differential gene sets were annotated in Kyoto Encyclopedia of Genes and Genomes database (KEGG) pathways to reveal information on differential gene functional potential and the carbohydrate-active enzymes (CAZy) database (http://www.cazy.org/) to obtain information on carbohydrate-active enzymes.

## Results and discussion

The modes of action of several yeast products vary due to differences in yeast strain, composition, processing effect, and yeast cell wall constituents. Therefore, comparisons of responses to yeast supplementation among studies require caution and clarification [6]. The product used in this study is a thermal-stable live *S. cerevisiae* (5.7 × 10^6^ colony forming units/g) with high levels of mannan oligosaccharides and beta-glucans, which were sourced from human-grade food plant (PMI, Arden Hills, MN, USA).

After sequencing of the 16 samples and assembly of the quality-controlled sequence data, 4,333,577 contigs, with an average read length of approximately 1,000 bp, were generated (see Additional file 1). Dietary yeast supplementation increased the relative abundance of 37 microbial species (see Additional file 2). The microbial species (> 1% of the total community) that responded to dietary treatment are shown in Table 1. *Ruminococcus albus* and *R. flavefaciens* are cellulose-degrading bacteria [15], *R. bromii* degrades resistant starch and xylan, while *R. obeum* degrades complex deoxy sugars, such as rhamnose and fucose [16]. Several studies have reported the effectiveness of certain strains of *S. cerevisiae* at increasing the abundance and activities of dominant fiber-degrading rumen microorganisms, such as Fibrobacter and *Ruminococcus* spp. [4]. This study also revealed that the abundance of relatively unknown and less abundant ruminal cellulolytic microbial species, such as *Rhodopseudomonas palustris* and *Sorangium cellulosum,* responded to YEA treatment (see Additional file 2). Some of the microbial species such as *R. obeum*, *Faecalibacterium prausnitzii*, and *Enterococcus casseliflavus*, that responded to YEA treatment are common human gut microbes and are not often detected in the rumen at high abundance (> 1%), therefore, further studies are needed to quantify these bacteria via quantitative polymerase chain reaction.

**Table 1:** Relative abundance of microbial species (> 1% of total sequences) affected by dietary treatment in beef steers fed no or 15 g/d of live yeast product

Microbial species	Treatment^a^		SE	*P*-value
	CON	YEA		
*Faecalibacterium prausnitzii*	0.83	1.45	0.08	0.01
*Ruminococcus flavefaciens*	0.28	1.21	0.13	0.01
*Oscillibacter valericigenes*	0.56	1.16	0.13	0.01
*Ruminococcus bromii*	0.70	1.66	0.18	0.01
*Methanobrevibacter ruminantium*	0.55	1.01	0.14	0.04
*Ruminococcus obeum*	0.35	1.22	0.10	0.01
*Enterococcus casseliflavus*	0.31	1.04	0.27	0.05
*Megasphaera elsdenii*	0.29	1.13	0.20	0.05
*Ruminococcus albus*	0.26	1.36	0.09	0.01

^a^CON = no yeast treatment; YEA = 15 g/d of live yeast product (PMI, Arden Hills, MN, USA)

Yeast additives maintain efficient rumen function necessary for the growth and activities of cellulolytic bacteria by increasing ruminal pH [1]. Yeast additives stimulate the growth of lactate-fermenting bacteria via supply of vitamins and amino acids essential for their growth [4] and/or outcompeting lactate-producers (such as *Streptococcus bovis*) for sugar utilization, thereby preventing ruminal lactate accumulation [1]. In this study, the relative abundance of *Megasphaera elsdenii*, a lactate-fermenting bacterium, and two species of *Desulfovibrio* (*D. desulfuricans* and *D. vulgaris*; see Additional file 2) known to oxidize lactate to acetate [17], were increased by YEA. It is important to note that lactate concentration was not influenced, probably because the diet fed was non-acidotic (as reported in our companion paper [7]).

The increased relative abundance of *Methanobrevibacter ruminantium*, a prominent member of methanogenic archaea, observed in this study is probably due to the increased abundance of fiber-degrading bacteria, especially *Ruminococcus albus,* a carbohydrate-fermenting, H_2_-producing organism [18]. This might be linked to H_2_ transfer between H_2_-producing cellulolytic bacteria and methanogenic archaea [19]. Fiber is fermented to volatile fatty acids, ammonia, H_2_, and CO_2_ in the rumen [20]. Rumen methanogens, such as *M. ruminantium*, use H_2_ as a substrate to reduce CO_2_ to methane [21]. Results of studies on the effects of strains of *S. cerevisiae* on ruminal methane production have been conflicting. An in vitro study by Chaucheyras-Durand et al. [22] revealed that a certain live yeast strain increased growth of acetogenic bacteria that can outcompete methanogens by utilizing H_2_ and CO_2_ to produce acetate. Newbold and Rode [23] evaluated the effects of several yeast strains on methane production and reported either no effect or decreased methane production. Our results suggest that YEA treatment might increase the amount of ruminal CH_4_ produced due to the increased abundance of *M. ruminantium*; however, we believe that CH_4_ production per unit of milk or meat will probably be reduced through improved ruminal fiber degradation and feed conversion efficiency.

To our knowledge, this study is the first to evaluate the influence of dietary treatment with yeast product on differentially abundant gene (DEG). A total of 154 DEGs were obtained (fold change > 2; FDR < 0.01). Exactly 139 genes were upregulated in the YEA treatment whereas 15 genes were upregulated in CON. The details of the DEGs, including gene IDs and statistical information, are shown in Additional file 3. None of the genes enriched in CON were annotated in KEGG. KEGG annotation analysis of the upregulated genes in YEA revealed that 10 pathways were enriched (Table 2). These 10 pathways were related to folding, sorting and degradation, translation, membrane transport, and metabolisms of vitamins and co-factors, energy, terpenoids and polyketides, amino acids, and carbohydrate pathways at KEGG orthology level 2 (Table 2). These confirm the results of our companion paper [6] in which we reported positive correlations between some bacterial genera that responded to yeast treatment and metabolites involved in metabolism of carbohydrates and amino acids. The DEGs related to carbohydrate metabolism were UDP-N-acetyl-2-amino-2-deoxyglucuronate dehydrogenase and β-N-acetylhexosaminidase, both of which are involved in metabolism of amino and nucleotide sugars, which are components of structural polysaccharides. The DEGs related to oxidative phosphorylation were ubiquinol-cytochrome c reductase cytochrome b subunit and cytochrome c oxidase subunit 2, both of which are components of the mitochondrial respiratory chain of *S. cerevisiae* that catalyzes the reduction of oxygen to water [24]. This indicates an oxygen-scavenging activity, one of the modes of action by which *S. cerevisiae* improves rumen function. Oxygen is toxic to rumen microbes; therefore, lowering the redox potential in the rumen improves the growing conditions for anaerobic rumen microbes [25]. This probably explains increased relative abundance of strictly anaerobic bacteria, such as *Oscillibacter valericigenes* and *F. prausnitzii*, observed in this study.

**Table 2:** KEGG orthology annotation of differential genes enriched in beef steers fed 15 g/d of live yeast product

KO level 2	KO level 3	KO level function
Carbohydrate metabolism	Amino sugar and nucleotide sugar metabolism [PATH: ko00520]	UDP-N-acetyl-2-amino-2-deoxyglucuronate dehydrogenase; Beta-N-acetylhexosaminidase
Energy metabolism	Oxidative phosphorylation [PATH: ko00190]	Ubiquinol-cytochrome c reductase cytochrome b subunit; Cytochrome c oxidase subunit 2.
Glycan biosynthesis and metabolism	Liposaccharide biosynthesis [PATH: ko00540]	3-deoxy-manno-octulosonate cytidylyltransferase
Metabolism of cofactors and vitamins	Pantothenate and CoA biosynthesis [PATH: ko00770]	Dephospho-CoA kinase; Pantoate--beta-alanine ligase
Metabolism of other amino acids	Glutathione metabolism [PATH: ko00480]	Aminopeptidase N
Metabolism of other amino acids	Beta-alanine metabolism [PATH: ko00410]	Pantoate--beta-alanine ligase
Metabolism of terpenoids and polyketides	Polyketide sugar unit biosynthesis [PATH: ko00523]	dTDP-4-dehydrorhamnose reductase
Folding, sorting and degradation	Protein export [PATH: ko03060]	Preprotein translocase subunit SecE
Translation	Ribosome [PATH: ko03010]	Large subunit ribosomal protein L15; Large subunit ribosomal protein L10
Membrane transport	Bacterial secretion system [PATH: ko03070]	Preprotein translocase subunit SecE

Lipopolysaccharides are components of gram-negative bacterial cell walls and are required for growth, virulence, and drug resistance of these bacteria [26]. The population of gram-negative bacteria accounts for up to 80%–90% of the total ruminal bacterial population [27]. In this study, increased lipopolysaccharide biosynthesis probably indicates improved ruminal bacterial growth. Feeding live yeast creates favorable conditions for growth and activities of ruminal bacteria through increased pH, lower redox potential, and supply of growth factors [25].

Rumen microbes are able to synthesize pantothenic acid and other B vitamins [28]; however, the net vitamin synthesis in the rumen may not be adequate to satisfy the nutritional requirements of the animals, especially in high-producing or growing animals [29]. *Saccharomyces cerevisiae* is capable of *de novo* biosynthesis of pantothenic acid and co-enzyme A via the activities of pantoate-beta-alanine ligase (using pantoate and alanine as substrates) and dephospho-CoA kinase (using dephospho-CoA as a substrate), respectively [30]. This explains the enriched pantothenic acid and CoA biosynthesis pathway and increased abundance of *Megasphaera elsdenii* and *Desulfovibrio* spp., in YEA treatment because lactate-utilizers require vitamins for their growth and activities [31]. Aminopeptidase N, also called alanyl aminopeptidase, is a hydrolase enzyme that catalyzes the cleavage of alanine from N-terminus of a dipeptide such as alanylglycine [32]. Enrichment of aminopeptidase N is probably due to increased demand of alanine as a substrate for pantothenic acid synthesis by *S. cerevisiae*.

Enrichment of pathways related to ribosome and protein export probably indicates increased microbial protein synthesis. Ribosomes are organelles that catalyze mRNA-directed protein synthesis in all organisms [33]. In our companion study, we observed lower ammonia-N in steers receiving YEA, which was partly due to its increased uptake by rumen microbes for protein synthesis. In this study, yeast supplementation improved rumen microbial growth, specifically the cellulolytic bacteria. Since cellulolytic bacteria have a high preference for ammonia-N [34], it was expected that utilization of ammonia-N by the rumen microbes for protein synthesis would increase.

**Fig. 1:**
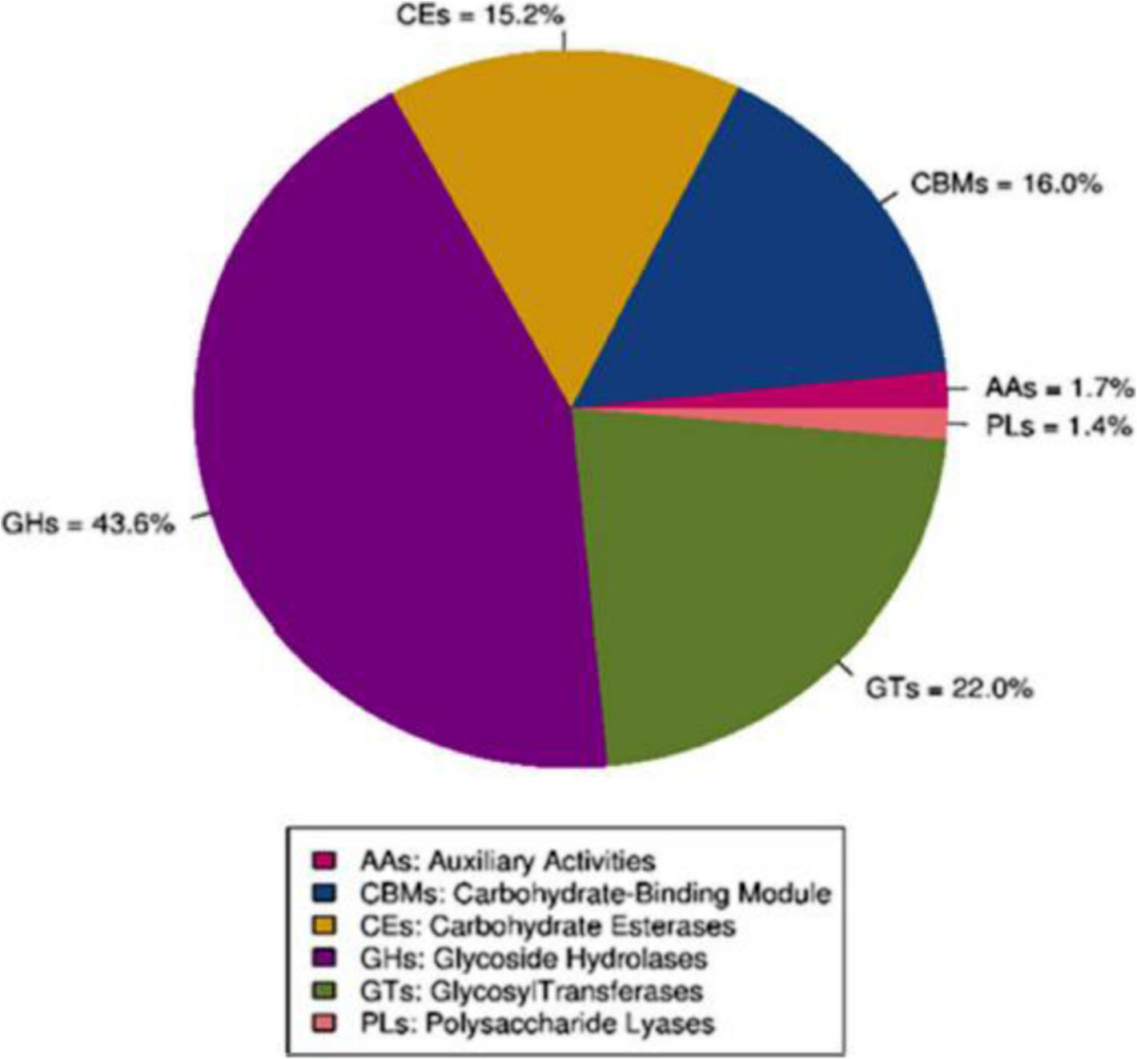
Distribution of the carbohydrate-active enzymes

The rumen contains several carbohydrate-active enzymes that are produced by rumen microorganisms and required for hydrolysis of plant cell-wall polysaccharides. A total of 148,586 predicted genes were annotated to the CAZy database, a database containing 6 classes of CAZy: glycoside hydrolases (GHs), glycosyltransferases (GTs), polysaccharide lyases (PLs), carbohydrate binding modules (CBM), carbohydrate esterases (CEs), and auxiliary activities (AAs). The distribution of the annotated genes is shown in Fig. 1. Annotation of the DEGs revealed that 3 genes belonged to GH3, GH16, and GH23 families; 4 genes belonged to GT2, GT4, GT9, and GT83 families; and 1 gene belonged to the CBM37 family (Table 3). Families of enzymes belonging to GHs and CBMs are polysaccharides-degrading enzymes produced by ruminal cellulolytic bacteria [35]. Increased abundance of GHs and CBMs was due to increased growth of cellulolytic bacteria in beef steer fed YEA. Glycosyltransferases represent a class of enzyme**s** involved in synthesizing glycosidic bonds for the biosynthesis of polysaccharides such as lipopolysaccharides [36]. This explains the enriched lipopolysaccharides biosynthesis pathway observed in steers fed YEA.

**Table 3:** Carbohydrate-active enzymes (CAZy) database annotation of differential genes enriched in beef steers fed 15 g/d of live yeast product

Family	CAZy-class^a^	Activities^b^
CBM37	CBM	Polysaccharide-degrading enzymes with broad specificity for xylan, chitin, microcrystalline and cellulose
GH16	GH	Xyloglucosyltransferase; endo-1,4-beta-galactosidase; endo-1,3-beta-glucanase; endo-1,3 (4)-beta-glucanase; licheninase; beta-agarase; carrageenase; xyloglucanase
GH23	GH	Peptidoglycan lyase
GH3	GH	Beta-glucosidase; xylan1,4-beta-xylosidase; beta-N-acetylhexosaminidase; 1,3-beta-glucosidase; 1,4-beta-glucosidase; exo-1,3-1,4-glucanase; alphalpha-L-arabinofuranosidase
GT2	GT	Cellulose synthase; chitin synthase; dolichyl-phosphate beta-*D*-mannosyltransferase; dolichyl-phosphate beta-glucosyltransferase; N-acetylglucosaminyltransferase; N-acetylgalactosaminyltransferase; hyaluronan synthase; chitin oligosaccharide synthase; beta-1,3-glucan synthase; beta-1,4-mannan synthase; alpha-1,6-mannosyltransferase; alpha-1,3-*L*-rhamnosyltransferase
GT4	GT	Sucrose synthase; sucrose-phosphate synthase; alpha-glucosyltransferase; lipopolysaccharide N-acetylglucosaminyltransferase; 1,2-diacylglycerol 3-glucosyltransferase; diglucosyl diacylglycerol synthase; digalactosyldiacylglycerol synthase
GT83	GT	4-amino-4-deoxy-beta-L-arabinosyltransferase; lipopolysaccharide core alpha-galacturonosyl transferase
GT9	GT	Lipopolysaccharide N-acetylglucosaminyltransferase; heptosyltransferase

^a^*CBM* Carbohydrate binding modules, *GH* glycoside hydrolases, *GT* Glycosyltransferases

^b^According to carbohydrate-active enzyme database (http://www.cazy.org/)

## Conclusion

This study provides new insights into the mode of action of live yeast, such as evidence of oxygen consumption and increased abundance of carbohydrate-active enzymes. Taken together, these results confirm the effectiveness of a live *S. cerevisiae* product at improving rumen function by increasing the abundance of cellulolytic bacteria, lactic acid-utilizing bacteria, and carbohydrate-active enzymes in the rumen.

## Additional files

Additional file 1: Details of sequence data. (XLSX 14 kb)

Additional file 2: Effects of live yeast supplementation on rumen microbial species. (DOCX 14 kb)

Additional file 3: List of differentially abundant genes. (XLSX 38 kb)

## Data Availability

All data generated or analyzed are available from the corresponding author on request.

## Abbreviations

bp: Base pair
CAZy: Carbohydrate-active enzymes
CD-HIT: Cluster database at high identity with tolerance
CON: Control treatment
DEG: Differentially abundant genes
DNA: Deoxyribonucleic acid
FDR: False discovery rate
KEGG: Kyoto Encyclopedia of Genes and Genomes
QUAST: Quality Assessment Tool for Genome Assemblies
rRNA: Ribosomal ribonucleic acid
YEA: Live yeast treatment
