# Supplementary material for: Effects of live yeast on differential genetic and functional attributes of rumen microbiota in beef cattle
Source: J Anim Sci Biotechnol. 2019 Sep 4;10:68. doi: 10.1186/s40104-019-0378-x (PMC6724239; doi:10.1186/s40104-019-0378-x)
Supplement: Supplementary file 2 — Effects of live yeast supplementation on rumen microbial species. (DOCX 14 kb) [file 40104_2019_378_MOESM2_ESM.docx]

|  | CON | YEA | *P*-value |
| --- | --- | --- | --- |
| *Rhodopseudomonas palustris* | 0.09 | 0.21 | 0.01 |
| *Enterococcus faecium* | 0.07 | 0.42 | 0.01 |
| *Sphingobacterium sp 21* | 0.06 | 0.15 | 0.01 |
| *Gordonibacter pamelaeae* | 0.11 | 0.24 | 0.01 |
| *Desulfovibrio_vulgaris* | 0.10 | 0.19 | 0.01 |
| *Ethanoligenens harbinense* | 0.18 | 0.32 | 0.01 |
| *Fretibacterium fastidiosum* | 0.08 | 0.15 | 0.01 |
| *Faecalibacterium prausnitzii* | 0.83 | 1.45 | 0.01 |
| *Ruminococcus*  *flavefaciens* | 0.28 | 1.21 | 0.01 |
| *Oscillibacter valericigenes* | 0.56 | 1.16 | 0.01 |
| *Slackia heliotrinireducens* | 0.18 | 0.32 | 0.01 |
| *Lawsonia_intracellularis* | 0.09 | 0.19 | 0.01 |
| *Ruminococcus bromii* | 0.70 | 1.66 | 0.01 |
| *Clostridium_sp_SY8519* | 0.17 | 0.31 | 0.01 |
| *Eubacterium_siraeum* | 0.15 | 0.24 | 0.01 |
| *Adlercreutzia_equolifaciens* | 0.14 | 0.25 | 0.02 |
| *Desulfovibrio desulfuricans* | 0.10 | 0.15 | 0.02 |
| *Symbiobacterium thermophilum* | 0.10 | 0.16 | 0.02 |
| *Mycobacterium_smegmatis* | 0.37 | 0.54 | 0.02 |
| *Eubacterium limosum* | 0.09 | 0.14 | 0.03 |
| *Bacteroides_fragilis* | 0.32 | 0.52 | 0.03 |
| *butyrate_producing_bacterium_SS3_4* | 0.12 | 0.19 | 0.03 |
| *Acidaminococcus fermentans* | 0.18 | 0.26 | 0.04 |
| *Candidatus Saccharimonas aalborgensis* | 0.07 | 0.15 | 0.04 |
| *Methanobrevibacter ruminantium* | 0.55 | 1.01 | 0.04 |
| *Enterococcus faecalis* | 0.06 | 0.15 | 0.04 |
| *Sorangium cellulosum* | 0.09 | 0.15 | 0.05 |
| *Rhodothermus marinus* | 0.09 | 0.14 | 0.05 |
| *Roseburia hominis* | 0.14 | 0.21 | 0.05 |
| *Ruminococcus obeum* | 0.35 | 1.22 | 0.01 |
| *Enterococcus casseliflavus* | 0.31 | 1.04 | 0.05 |
| *Olsenella uli* | 0.20 | 0.41 | 0.05 |
| *Megasphaera elsdenii* | 0.29 | 1.13 | 0.05 |
| *Bifidobacterium bifidum* | 0.08 | 0.15 | 0.06 |
| *Ruminococcus albus* | 0.26 | 1.36 | 0.01 |
| *Porphyromonas asaccharolytica* | 0.14 | 0.20 | 0.08 |
| *Roseburia_intestinalis* | 0.12 | 0.17 | 0.08 |

Additional file 2: Table S2. Effects of live yeast supplementation on rumen microbial species
